# [Corrigendum] Use of methylation profiling to identify significant differentially methylated genes in bone marrow mesenchymal stromal cells from acute myeloid leukemia

**DOI:** 10.3892/ijmm.2025.5520

**Published:** 2025-03-17

**Authors:** Jing Huang, Zhi Liu, Yufan Sun, Qi Zhong, Li Xu, Ruimin Ou, Cheng Li, Rui Chen, Mengdong Yao, Qing Zhang, Shuang Liu

Int J Mol Med 41: 679-686, 2018; DOI: 10.3892/ijmm.2017.3271

Following the publication of the above article, an interested reader drew to the authors' and the Editor's attention that, given the subject matter of the article, in a few places in the text throughout the paper, 'MIR159' was probably intended to have been written as 'MIR596', and 'miR-159' was probably meant to have been written as 'miR-596'.

The authors have responded to confirm that the interested reader was correct in their assessment; this paper discusses the role of miR-596 in the context of acute myeloid leukemia (AML) and its potential as a biomarker, and this error appears to have occurred during the final stages of manuscript preparation. Therefore, the following corrections to the text of this article should be noted:

- In the final sentence of the Abstract on p. 679, 'miR-596' should have been written, rather than 'miR-159'. The corrected sentence should read as follows: 'Furthermore, the aberrantly hypermethylated **miR-596**-encoding gene ***MIR596*** may be a potential biomarker of AML.'
- In the Discussion, all instances where 'miR-159' and 'MIR159' were mentioned should be corrected to 'miR-596' and 'MIR596', respectively. The sentences concerned should have read as follows (p. 685, right-hand column, line 9): 'Thus, it was suggested that the hypermethylation of **miR-596** may be associated with its transcriptional regulation. It was also inferred that **miR-596** methylation in BM-MSCs may be a biomarker or prognostic factor for patients with AML. However, the significance of the methylation of **miR-596** demands further investigation." In addition, the sentence starting on p. 685, right-hand column, second paragraph, line 5, should have read as follows: 'Furthermore, the aberrant hypermethylated **miR-596**-encoding gene ***MIR596*** may be a potential biomarker of AML.'

All the authors agree with the publication of this corrigendum; furthermore, they also apologize to the readership of the journal for any inconvenience caused.

